# Therapeutical Notes

**Published:** 1898-12-31

**Authors:** 


### THERAPEUTICAL NOTES.

### Itching in Jaundice.

The following is recommended by Boullard for this troublesome affection:—

℞ Ichthol 5·0—10· gme.
Alcohol.
Ether, āā 50 gme.

M. S.—To be rubbed in several times a day.

*Ges. Therapic.*, p. 380, 1898.

